# The genome sequence of the Northern Bottlenose Whale, *Hyperoodon ampullatus *(Forster, 1770)

**DOI:** 10.12688/wellcomeopenres.22743.1

**Published:** 2024-07-26

**Authors:** Laura Joan Feyrer, Evelien de Greef, Wellcome Sanger Institute Tree of Life Management, Samples and Laboratory team, Wellcome Sanger Institute Scientific Operations: Sequencing Operations, Wellcome Sanger Institute Tree of Life Core Informatics team, Tree of Life Core Informatics collective, Darwin Tree of Life Consortium

**Affiliations:** 1 Dalhousie University, Halifax, Nova Scotia, Canada; 2 University of Manitoba, Winnipeg, Manitoba, Canada

**Keywords:** Hyperoodon ampullatus, Northern Bottlenose Whale, genome sequence, chromosomal, Artiodactyla

## Abstract

We present a genome assembly from an individual female *Hyperoodon ampullatus* (the Northern Bottlenose Whale; Chordata; Mammalia; Artiodactyla; Ziphiidae). The genome sequence spans 2,828.70 megabases. Most of the assembly is scaffolded into 21 chromosomal pseudomolecules, including the X sex chromosome. The mitochondrial genome has also been assembled and is 16.34 kilobases in length.

## Species taxonomy

Eukaryota; Opisthokonta; Metazoa; Eumetazoa; Bilateria; Deuterostomia; Chordata; Craniata; Vertebrata; Gnathostomata; Teleostomi; Euteleostomi; Sarcopterygii; Dipnotetrapodomorpha; Tetrapoda; Amniota; Mammalia; Theria; Eutheria; Boreoeutheria; Laurasiatheria; Artiodactyla; Whippomorpha; Cetacea; Odontoceti; Ziphiidae; *Hyperoodon*; *Hyperoodon ampullatus* (Forster, 1770) (NCBI:txid48744).

## Background

**Figure 1.**
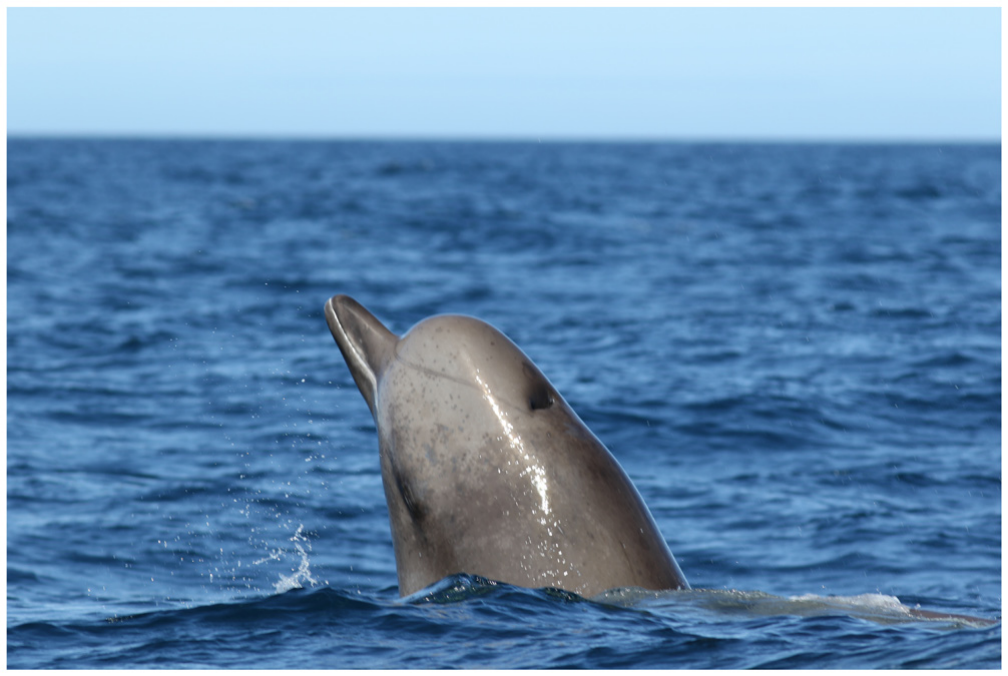
Photograph of *Hyperoodon ampullatus* (not the specimen used for genome sequencing).

Northern bottlenose whales ( *Hyperoodon ampullatus*) ( Figure 1) are one of two species in the genus *Hyperoodon* and one of the most well-known beaked whale species in the diverse family Ziphiidae. Having a robust light grey to brown dolphin-like appearance, they can be distinguished by their substantially larger size, a curved dorsal fin located far back on their body, small blunt flippers, and tail flukes that lack a central notch. Northern bottlenose whales are characterised by their prominent melons (foreheads), and as they age, a whale’s melon will lighten, becoming white in older individuals. Mature males can reach 9 m in length, over a metre longer than adult females. Sexual dimorphism in physical size, pronounced square-shaped melons, large maxillary skull crests, and two small erupted teeth, led early taxonomists to initially suggest the sexes were two separate species of beaked whales ( Alves *et al.*, 2023; Ellis & Mead, 2017; Gol’din, 2014). While sexual display is prevalent among beaked whale species, male northern bottlenose whales are unique in that they use their enlarged melons and reinforced skull morphology in ritualised head-butting contests ( Gowans & Rendell, 1999).

Residing only in the deep waters of the northern North Atlantic, our understanding of northern bottlenose whale biology is largely due to a history of commercial exploitation, where whalers first hunted them for their rich oil and then, in the post-war era, for the pet food market ( NAMMCO, 2018). Their social behaviour and tendency to approach vessels made them easy targets, allowing whalers to kill them in large numbers ( Whitehead & Hooker, 2012). Due to the significant impacts of whaling across their populations, and ongoing threats from human impacts ( Feyrer *et al.*, 2024b), they are recognised as a species at risk in Canada and are considered Near Threatened by the IUCN Red List assessment ( IUCN, 2022).

Like other beaked whale species, northern bottlenose whales are specialised foragers, who use echolocation to hunt for squid while diving to extreme depths (800–1400 m) for upwards of an hour ( Hooker & Baird, 1999). Sightings, acoustic detections, and species distribution models indicate that northern bottlenose whale habitat is concentrated along the edge of continental shelves, at depths averaging around 1200 m ( Feyrer *et al.*, 2024a). Post-whaling, a small remnant population inhabiting submarine canyons at the southern edge of their range off Nova Scotia, Canada, has been the subject of the longest ecological study of any beaked whale species ( Hooker *et al.*, 2019). Due to their energetic demands ( New *et al.*, 2013), need for predictable resource stability ( Gowans *et al.*, 2007), and social learning associated with finding and localising prey ( Whitehead, 2010), northern bottlenose whales exhibit high site fidelity to the Gully submarine canyon, with repeated sightings of the same individuals over 30 years ( Feyrer *et al.*, 2021). In 2004, the Gully became a national marine protected area, and the protection of northern bottlenose whales is a key conservation objective.

Here we present a chromosomally complete genome sequence for the northern bottlenose whale, which was sequenced as part of the Vertebrate Genomes Project and the Darwin Tree of Life project. Genomic research on the northern bottlenose whale will open avenues to better understand patterns of genome-wide variation in marine mammals, overcoming previous limitations due to a fragmented draft assembly ( de Greef *et al.*, 2022). Goals for analysing this genome include investigating the recent demographic history to assess the impact of historical whaling on genetic diversity (e.g., Santiago *et al.*, 2020) and delineating subtle population structure patterns in northern bottlenose whales, which are currently unclear ( de Greef *et al.*, 2022). Additionally, the northern bottlenose whale genome will guide studies on local adaptation across the species’ large latitudinal range and potential mechanisms behind phenotypic and physiological traits. This resource is expected to support comparative genomic studies among marine mammals, leading to advances in our understanding of diving physiology ( Hindle, 2020) and cancer biology ( Tollis *et al.*, 2019).

## Genome sequence report

The genome of an adult female *Hyperoodon ampullatus* was sequenced using Pacific Biosciences single-molecule HiFi long reads, generating a total of 22.21 Gb (gigabases) from 2.67 million reads, providing approximately 28-fold coverage. Primary assembly contigs were scaffolded with chromosome conformation Hi-C data, which produced 464.04 Gbp from 3,073.09 million reads, yielding an approximate coverage of 164-fold. Specimen and sequencing information is summarised in Table 1.

**Table 1.** Specimen and sequencing data for *Hyperoodon ampullatus*.

Project information			
**Study title**	Hyperoodon ampullatus (northern bottlenose whale)		
**Umbrella BioProject**	PRJEB60010		
**Species**	*Hyperoodon ampullatus*		
**BioSample**	SAMEA10839125		
**NCBI taxonomy ID**	48744		
Specimen information			
Technology	ToLID	BioSample accession	Organism part
**PacBio long read sequencing**	mHypAmp2	SAMEA10839128	skin
**Hi-C sequencing**	mHypAmp1	SAMEA10839127	skin
**RNA sequencing**	mHypAmp3	SAMEA10839129	skin
Sequencing information			
Platform	Run accession	Read count	Base count (Gb)
**Hi-C Illumina NovaSeq 6000**	ERR10908646	3.07e+09	464.04
**PacBio Sequel IIe**	ERR10906107	2.60e+06	20.13
**PacBio Sequel IIe**	ERR10906109	3.03e+06	24.86
**PacBio Sequel IIe**	ERR10906108	2.40e+06	23.05
**PacBio Sequel IIe**	ERR10906110	2.67e+06	22.21
**RNA Illumina NovaSeq 6000**	ERR11837470	4.21e+07	6.36

**Figure 2.**
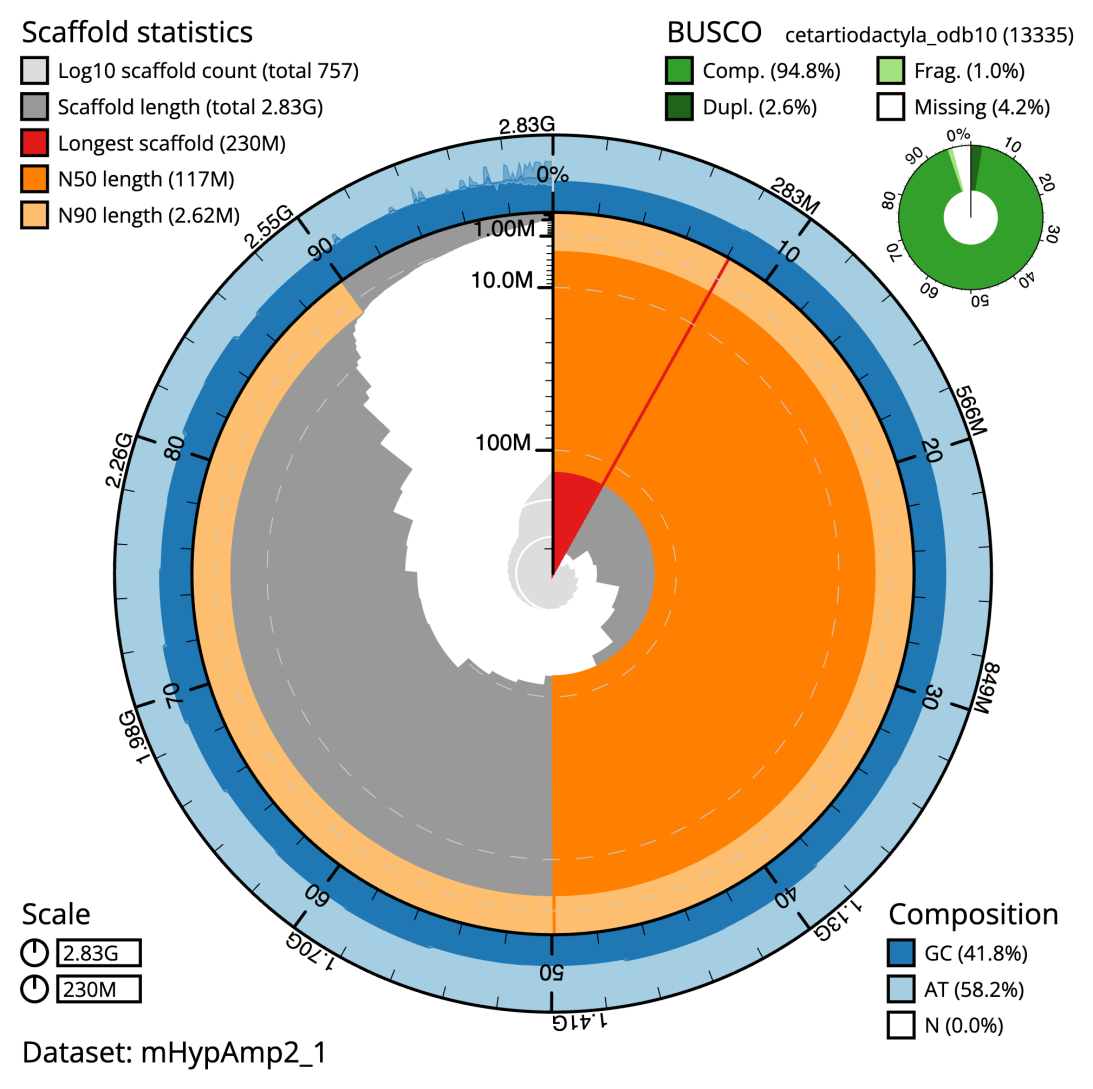
Genome assembly of *Hyperoodon ampullatus*, mHypAmp2.1: metrics. The BlobToolKit snail plot shows N50 metrics and BUSCO gene completeness. The main plot is divided into 1,000 size-ordered bins around the circumference with each bin representing 0.1% of the 2,828,760,712 bp assembly. The distribution of scaffold lengths is shown in dark grey with the plot radius scaled to the longest scaffold present in the assembly (230,449,670 bp, shown in red). Orange and pale-orange arcs show the N50 and N90 scaffold lengths (117,448,692 and 2,622,535 bp), respectively. The pale grey spiral shows the cumulative scaffold count on a log scale with white scale lines showing successive orders of magnitude. The blue and pale-blue area around the outside of the plot shows the distribution of GC, AT and N percentages in the same bins as the inner plot. A summary of complete, fragmented, duplicated and missing BUSCO genes in the cetartiodactyla_odb10 set is shown in the top right. An interactive version of this figure is available at https://blobtoolkit.genomehubs.org/view/Hyperoodon%20ampullatus/dataset/mHypAmp2_1/snail.

**Figure 3.**
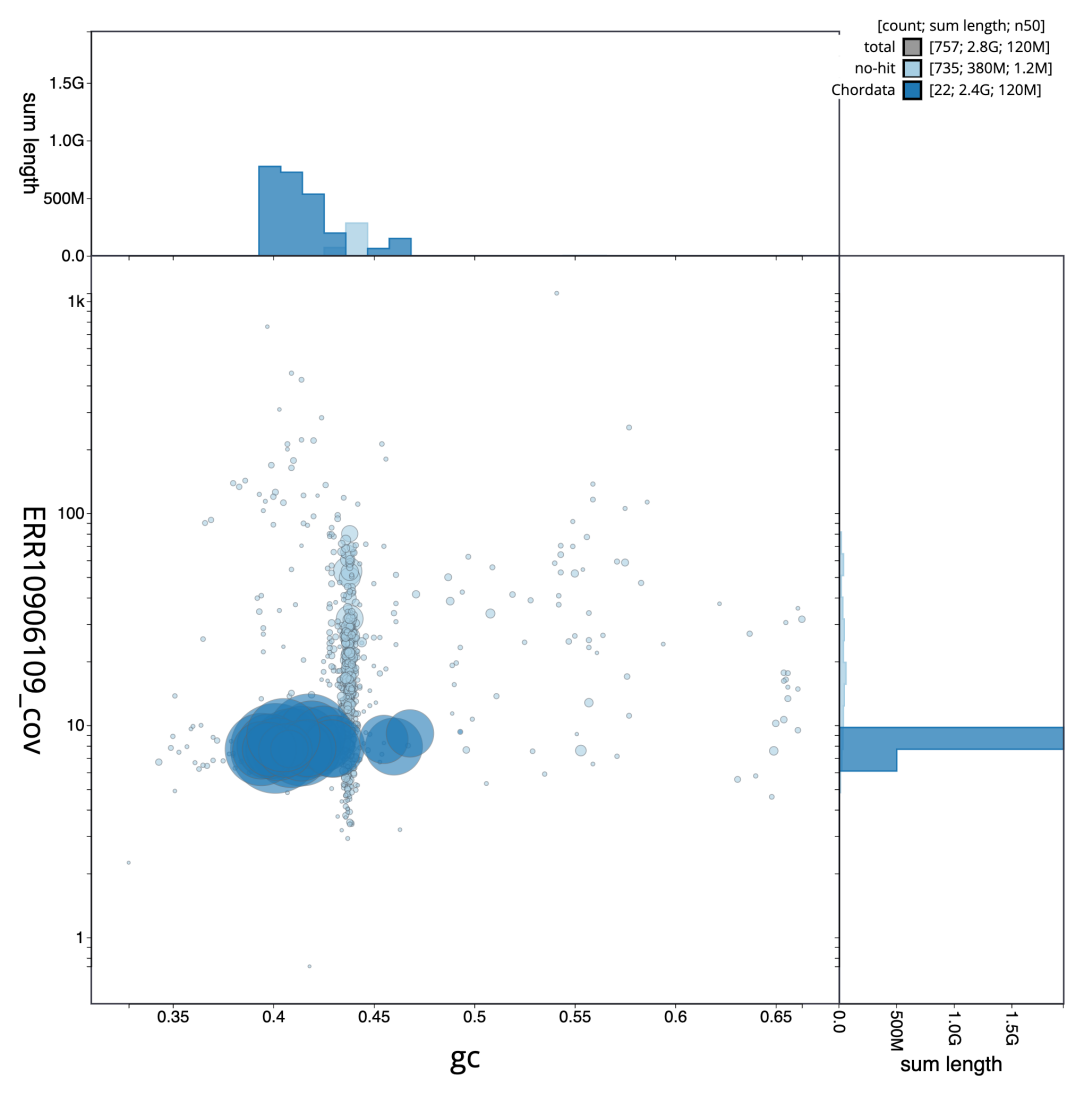
Genome assembly of *Hyperoodon ampullatus*, mHypAmp2.1: BlobToolKit GC-coverage plot. Sequences are coloured by phylum. Circles are sized in proportion to sequence length. Histograms show the distribution of sequence length sum along each axis. An interactive version of this figure is available at https://blobtoolkit.genomehubs.org/view/Hyperoodon%20ampullatus/dataset/mHypAmp2_1/blob.

**Figure 4.**
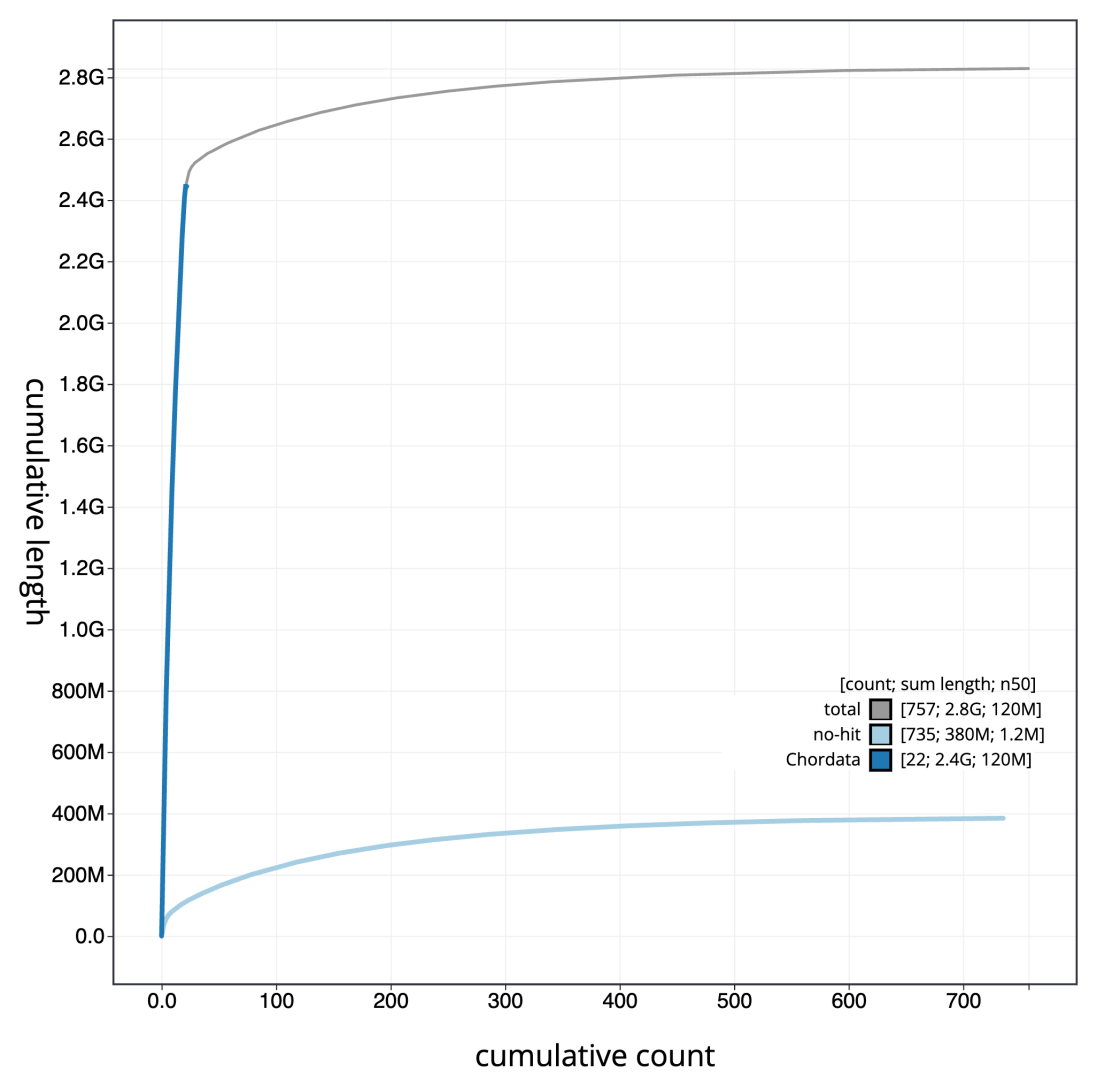
Genome assembly of *Hyperoodon ampullatus* mHypAmp2.1: BlobToolKit cumulative sequence plot. The grey line shows cumulative length for all sequences. Coloured lines show cumulative lengths of sequences assigned to each phylum using the buscogenes taxrule. An interactive version of this figure is available at https://blobtoolkit.genomehubs.org/view/Hyperoodon%20ampullatus/dataset/mHypAmp2_1/cumulative.

**Figure 5.**
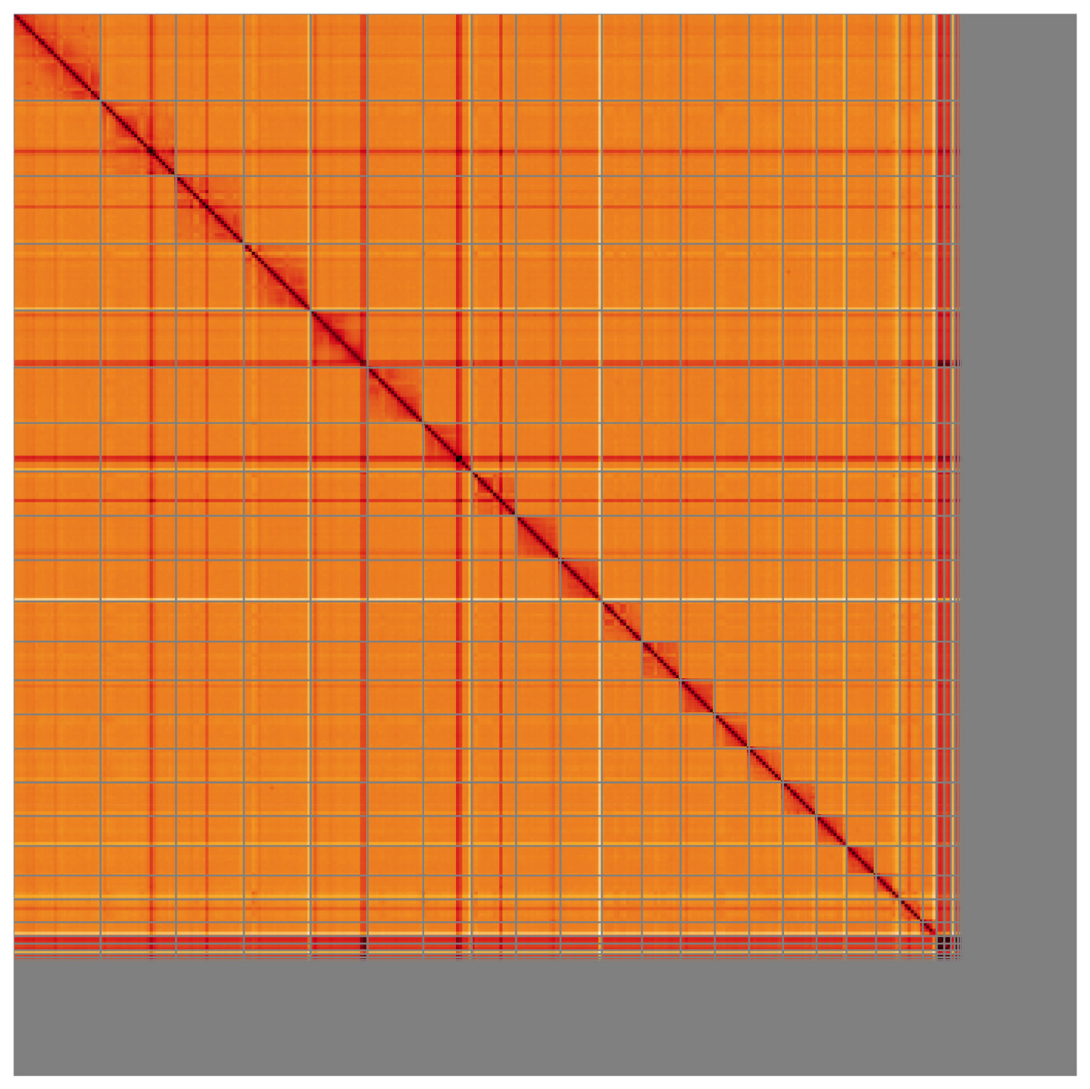
Genome assembly of *Hyperoodon ampullatus* mHypAmp2.1: Hi-C contact map of the mHypAmp2.1 assembly, visualised using HiGlass. Chromosomes are shown in order of size from left to right and top to bottom. An interactive version of this figure may be viewed at https://genome-note-higlass.tol.sanger.ac.uk/l/?d=bSkraM71TtCcL_m9Em5AFw.

Manual assembly curation corrected 112 missing joins or mis-joins and one haplotypic duplication, reducing the scaffold number by 8.69%, and increasing the scaffold N50 by 10.37%. The final assembly has a total length of 2,828.70 Mb in 756 sequence scaffolds with a scaffold N50 of 117.4 Mb ( Table 2). The snail plot in Figure 2 provides a summary of the assembly statistics, while the distribution of assembly scaffolds on GC proportion and coverage is shown in Figure 3. The cumulative assembly plot in Figure 4 shows curves for subsets of scaffolds assigned to different phyla. Most (89.22%) of the assembly sequence was assigned to 21 chromosomal-level scaffolds, representing 20 autosomes and the X sex chromosome. Chromosome-scale scaffolds confirmed by the Hi-C data are named in order of size ( Figure 5; Table 3). The X chromosome was identified based on alignment with the *Mesoplodon densirostris* genome assembly (GCA_025265405.1). The order and orientation of contigs in the following regions is uncertain: Chromosome 2, 131 Mb to 140 Mb; Chromosome 6, 91 Mb to 102 Mb and Chromosome 7, 76 Mb to 81 Mb. While not fully phased, the assembly deposited is of one haplotype. Contigs corresponding to the second haplotype have also been deposited. The mitochondrial genome was also assembled and can be found as a contig within the multifasta file of the genome submission.

**Table 2.** Genome assembly data for *Hyperoodon ampullatus*, mHypAmp2.1.

Genome assembly		
Assembly name	mHypAmp2.1	
Assembly accession	GCA_949752795.1	
*Accession of alternate haplotype*	*GCA_949752845.1*	
Span (Mb)	2,828.70	
Number of contigs	1,855	
Contig N50 length (Mb)	3.1	
Number of scaffolds	756	
Scaffold N50 length (Mb)	117.4	
Longest scaffold (Mb)	230.86	
Assembly metrics *		*Benchmark*
Consensus quality (QV)	61.9	*≥ 50*
*k*-mer completeness	100.0%	*≥ 95%*
BUSCO **	C:94.9%[S:92.3%,D:2.6%],F:1.0%,M:4.1%,n:13,335	*C ≥ 95%*
Percentage of assembly mapped to chromosomes	89.22%	*≥ 95%*
Sex chromosomes	X	*localised homologous pairs*
Organelles	Mitochondrial genome: 16.34 kb	*complete single alleles*

* Assembly metric benchmarks are adapted from column VGP-2020 of “Table 1: Proposed standards and metrics for defining genome assembly quality” from Rhie *et al.* (2021). ** BUSCO scores based on the cetartiodactyla_odb10 BUSCO set using version 5.3.2. C = complete [S = single copy, D = duplicated], F = fragmented, M = missing, n = number of orthologues in comparison. A full set of BUSCO scores is available at https://blobtoolkit.genomehubs.org/view/Hyperoodon%20ampullatus/dataset/mHypAmp2_1/busco.

**Table 3.** Chromosomal pseudomolecules in the genome assembly of *Hyperoodon ampullatus*, mHypAmp2.

INSDC accession	Name	Length (Mb)	GC%
OX457056.1	1	230.45	40.0
OX457057.1	2	199.77	42.0
OX457058.1	3	179.68	41.5
OX457059.1	4	176.6	41.0
OX457060.1	5	147.16	39.5
OX457061.1	6	128.22	42.0
OX457062.1	7	117.53	42.5
OX457063.1	8	117.45	40.0
OX457064.1	9	109.45	40.0
OX457065.1	10	106.41	43.0
OX457066.1	11	102.38	41.5
OX457067.1	12	90.65	39.5
OX457068.1	13	90.64	43.0
OX457069.1	14	89.65	46.0
OX457070.1	15	88.92	41.5
OX457071.1	16	79.6	40.0
OX457072.1	17	78.29	40.5
OX457073.1	18	63.07	45.5
OX457074.1	19	60.68	47.0
OX457075.1	20	36.58	41.0
OX457055.1	X	151.25	40.5
OX457076.1	MT	0.02	39.5

The estimated Quality Value (QV) of the final assembly is 61.9 with *k*-mer completeness of 100.0%, and the assembly has a BUSCO v5.3.2 completeness of 94.9% (single = 92.3%, duplicated = 2.6%), using the cetartiodactyla_odb10 reference set ( *n* = 13,335).

Metadata for specimens, BOLD barcode results, spectra estimates, sequencing runs, contaminants and pre-curation assembly statistics are given at https://links.tol.sanger.ac.uk/species/48744.

## Methods

### Sample acquisition and nucleic acid extraction

The samples used were collected from the Gully Marine Protected Area, Nova Scotia, Canada (latitude 43.84, longitude –58.91) on 2019-08-06. Samples were taken from three different live animals by biopsy with a crossbow. A sample from an adult female *Hyperoodon ampullatus* (specimen ID SAN00001899, ToLID mHypAmp2) was used for genome sequencing. Another sample was used for Hi-C sequencing (specimen ID SAN00001898, ToLID mHypAmp1) and a third for RNA sequencing (specimen ID SAN00001900, ToLID mHypAmp3). The specimens were collected by Laura Feyrer (Dalhousie University) and preserved by snap freezing.

The workflow for high molecular weight (HMW) DNA extraction at the Wellcome Sanger Institute (WSI) Tree of Life Core Laboratory includes a sequence of core procedures: sample preparation; sample homogenisation, DNA extraction, fragmentation, and clean-up. In sample preparation, the mHypAmp2 sample was weighed and dissected on dry ice ( Jay *et al.*, 2023).

Tissue from the skin was homogenised using a PowerMasher II tissue disruptor ( Denton *et al.*, 2023a). HMW DNA was extracted using the Automated MagAttract v1 protocol ( Sheerin *et al.*, 2023). DNA was sheared into an average fragment size of 12–20 kb in a Megaruptor 3 system with speed setting 30 ( Todorovic *et al.*, 2023). Sheared DNA was purified by solid-phase reversible immobilisation ( Strickland *et al.*, 2023): in brief, the method employs a 1.8X ratio of AMPure PB beads to sample to eliminate shorter fragments and concentrate the DNA. The concentration of the sheared and purified DNA was assessed using a Nanodrop spectrophotometer and Qubit Fluorometer using the Qubit dsDNA High Sensitivity Assay kit. Fragment size distribution was evaluated by running the sample on the FemtoPulse system.

RNA was extracted from skin tissue of mHypAmp3 in the Tree of Life Laboratory at the WSI using the RNA Extraction: Automated MagMax™ *mir*Vana protocol ( do Amaral *et al.*, 2023). The RNA concentration was assessed using a Nanodrop spectrophotometer and a Qubit Fluorometer using the Qubit RNA Broad-Range Assay kit. Analysis of the integrity of the RNA was done using the Agilent RNA 6000 Pico Kit and Eukaryotic Total RNA assay.

Protocols developed by the WSI Tree of Life laboratory are publicly available on protocols.io ( Denton *et al.*, 2023b).

### Sequencing

Pacific Biosciences HiFi circular consensus DNA sequencing libraries were constructed according to the manufacturers’ instructions. Poly(A) RNA-Seq libraries were constructed using the NEB Ultra II RNA Library Prep kit. DNA and RNA sequencing was performed by the Scientific Operations core at the WSI on Pacific Biosciences Sequel IIe (HiFi) and Illumina NovaSeq 6000 (RNA-Seq) instruments. Hi-C data were also generated from skin tissue of mHypAmp1 using the Arima-HiC v2 kit. The Hi-C sequencing was performed using paired-end sequencing with a read length of 150 bp on the Illumina NovaSeq 6000 instrument.

### Genome assembly, curation and evaluation

***Assembly.*** Original assembly of HiFi reads was performed using Hifiasm ( Cheng *et al.*, 2021) with the --primary option. Haplotypic duplications were identified and removed with purge_dups ( Guan *et al.*, 2020). Hi-C reads are further mapped with bwa-mem2 ( Vasimuddin *et al.*, 2019) to the primary contigs, which are further scaffolded using the provided Hi-C data ( Rao *et al.*, 2014) in YaHS ( Zhou *et al.*, 2023) using the --break option. Scaffolded assemblies are evaluated using Gfastats ( Formenti *et al.*, 2022), BUSCO ( Manni *et al.*, 2021) and MERQURY.FK ( Rhie *et al.*, 2020). The mitochondrial genome was assembled using MitoHiFi ( Uliano-Silva *et al.*, 2023), which runs MitoFinder ( Allio *et al.*, 2020) and uses these annotations to select the final mitochondrial contig and to ensure the general quality of the sequence.

***Assembly curation.*** The assembly was decontaminated using the Assembly Screen for Cobionts and Contaminants (ASCC) pipeline (article in preparation). Manual curation was primarily conducted using PretextView ( Harry, 2022), with additional insights provided by JBrowse2 ( Diesh *et al.*, 2023) and HiGlass ( Kerpedjiev *et al.*, 2018). Scaffolds were visually inspected and corrected as described by Howe *et al*. (2021). Any identified contamination, missed joins, and mis-joins were corrected, and duplicate sequences were tagged and removed. The X chromosome was identified based on synteny. The entire process is documented at https://gitlab.com/wtsi-grit/rapid-curation (article in preparation).

### Evaluation of the final assembly

A Hi-C map for the final assembly was produced using bwa-mem2 ( Vasimuddin *et al.*, 2019) in the Cooler file format ( Abdennur & Mirny, 2020). To assess the assembly metrics, the *k*-mer completeness and QV consensus quality values were calculated in Merqury ( Rhie *et al.*, 2020). This work was done using Nextflow ( Di Tommaso *et al.*, 2017) DSL2 pipelines “sanger-tol/readmapping” ( Surana *et al.*, 2023a) and “sanger-tol/genomenote” ( Surana *et al.*, 2023b). The genome was analysed within the BlobToolKit environment ( Challis *et al.*, 2020) and BUSCO scores ( Manni *et al.*, 2021; Simão *et al.*, 2015) were calculated.

The evaluation pipelines were developed using the nf-core tooling ( Ewels *et al.*, 2020), use MultiQC ( Ewels *et al.*, 2016), and make extensive use of the Conda package manager, the Bioconda initiative ( Grüning *et al.*, 2018), the Biocontainers infrastructure ( da Veiga Leprevost *et al.*, 2017), and the Docker ( Merkel, 2014) and Singularity ( Kurtzer *et al.*, 2017) containerisation solutions. Table 4 contains a list of relevant software tool versions and sources.

**Table 4.** Software tools: versions and sources.

Software tool	Version	Source
BEDTools	2.30.0	https://github.com/arq5x/bedtools2
BLAST	2.14.0	ftp://ftp.ncbi.nlm.nih.gov/blast/executables/blast+/
BlobToolKit	4.3.7	https://github.com/blobtoolkit/blobtoolkit
BUSCO	5.3.2 and 5.5.0	https://gitlab.com/ezlab/busco
bwa-mem2	2.2.1	https://github.com/bwa-mem2/bwa-mem2
Cooler	0.8.11	https://github.com/open2c/cooler
DIAMOND	2.1.8	https://github.com/bbuchfink/diamond
fasta_windows	0.2.4	https://github.com/tolkit/fasta_windows
FastK	427104ea91c78c3b8b8b49f1a7d6bbeaa869ba1c	https://github.com/thegenemyers/FASTK
Gfastats	1.3.6	https://github.com/vgl-hub/gfastats
Hifiasm	0.16.1-r375	https://github.com/chhylp123/hifiasm
HiGlass	44086069ee7d4d3f6f3f0012569789ec138f42b84 aa44357826c0b6753eb28de	https://github.com/higlass/higlass
Merqury.FK	d00d98157618f4e8d1a9190026b19b471055b22e	https://github.com/thegenemyers/MERQURY.FK
MitoHiFi	2	https://github.com/marcelauliano/MitoHiFi
MultiQC	1.14, 1.17, and 1.18	https://github.com/MultiQC/MultiQC
Nextflow	23.04.0-5857	https://github.com/nextflow-io/nextflow
PretextView	0.2	https://github.com/sanger-tol/PretextView
purge_dups	1.2.3	https://github.com/dfguan/purge_dups
samtools	1.16.1, 1.17, and 1.18	https://github.com/samtools/samtools
sanger-tol/ ascc	-	https://github.com/sanger-tol/ascc
sanger-tol/ genomenote	1.1.1	https://github.com/sanger-tol/genomenote
sanger-tol/readmapping	1.2.1	https://github.com/sanger-tol/readmapping
Singularity	3.9.0	https://github.com/sylabs/singularity
YaHS	1.2a	https://github.com/c-zhou/yahs

Table 4 contains a list of relevant software tool versions and sources.

### Wellcome Sanger Institute – Legal and Governance

The materials that have contributed to this genome note have been supplied by a Darwin Tree of Life Partner. The submission of materials by a Darwin Tree of Life Partner is subject to the **‘Darwin Tree of Life Project Sampling Code of Practice’**, which can be found in full on the Darwin Tree of Life website here. By agreeing with and signing up to the Sampling Code of Practice, the Darwin Tree of Life Partner agrees they will meet the legal and ethical requirements and standards set out within this document in respect of all samples acquired for, and supplied to, the Darwin Tree of Life Project.

Further, the Wellcome Sanger Institute employs a process whereby due diligence is carried out proportionate to the nature of the materials themselves, and the circumstances under which they have been/are to be collected and provided for use. The purpose of this is to address and mitigate any potential legal and/or ethical implications of receipt and use of the materials as part of the research project, and to ensure that in doing so we align with best practice wherever possible. The overarching areas of consideration are:

• Ethical review of provenance and sourcing of the material

• Legality of collection, transfer and use (national and international)

Each transfer of samples is further undertaken according to a Research Collaboration Agreement or Material Transfer Agreement entered into by the Darwin Tree of Life Partner, Genome Research Limited (operating as the Wellcome Sanger Institute), and in some circumstances other Darwin Tree of Life collaborators.

## Data Availability

European Nucleotide Archive: *Hyperoodon ampullatus* (northern bottlenose whale). Accession number PRJEB60010; https://identifiers.org/ena.embl/PRJEB60010 ( Wellcome Sanger Institute, 2023). The genome sequence is released openly for reuse. The *Hyperoodon ampullatus* genome sequencing initiative is part of the Darwin Tree of Life (DToL) Project and Vertebrate Genomes Project. All raw sequence data and the assembly have been deposited in INSDC databases. The genome will be annotated using available RNA-Seq data and presented through the Ensembl pipeline at the European Bioinformatics Institute. Raw data and assembly accession identifiers are reported in Table 1 and Table 2.

## Author information

Members of the Wellcome Sanger Institute Tree of Life Management, Samples and Laboratory team are listed here: https://doi.org/10.5281/zenodo.12162482.

Members of Wellcome Sanger Institute Scientific Operations: Sequencing Operations are listed here: https://doi.org/10.5281/zenodo.12165051.

Members of the Wellcome Sanger Institute Tree of Life Core Informatics team are listed here: https://doi.org/10.5281/zenodo.12160324.

Members of the Tree of Life Core Informatics collective are listed here: https://doi.org/10.5281/zenodo.12205391.

Members of the Darwin Tree of Life Consortium are listed here: https://doi.org/10.5281/zenodo.4783558.
