# Supplementary material for: The genome sequence of the Northern Bottlenose Whale, Hyperoodon ampullatus (Forster, 1770)
Source: Wellcome Open Res. 2024 Jul 26;9:410. [Version 1] doi: 10.12688/wellcomeopenres.22743.1 (PMC11621617; doi:10.12688/wellcomeopenres.22743.1)
Supplement: Peer review history

# Reviewer report: Reviewer response for version 1

**DOI:** 10.21956/wellcomeopenres.25046.r112893

**Published:** 2024-12-05

**Authors:** Mariana F Nery

**Affiliations:** 1 Universidade Estadual de Campinas, São Paulo, Brazil

This article describes the sequencing and assembly of a chromosomal-level genome for the Northern Bottlenose Whale ( *Hyperoodon ampullatus*), providing a comprehensive overview of the data and methods used. The well-written introduction sets the context for the genomic data by addressing species biology, conservation concerns, and potential applications. The methods are clearly described and technically sound.

 The datasets are presented in a clear and accessible format, and the assembly quality metrics meet high standards. 

 Besides the points the first reviewer raised, and with whom I agree, the only additional suggestion is to consider omitting the methods related to RNA sequencing in this particular report. As the RNA data will be used for annotation, and annotation is not addressed in this specific article, these methods could be more appropriately described in future indexing.

Are sufficient details of methods and materials provided to allow replication by others?

Yes

Is the rationale for creating the dataset(s) clearly described?

Yes

Are the datasets clearly presented in a useable and accessible format?

Yes

Are the protocols appropriate and is the work technically sound?

Yes

Reviewer Expertise:

Genomics and molecular evolution

I confirm that I have read this submission and believe that I have an appropriate level of expertise to confirm that it is of an acceptable scientific standard.

# Reviewer report: Reviewer response for version 1

**DOI:** 10.21956/wellcomeopenres.25046.r97205

**Published:** 2024-09-23

**Authors:** Phillip Morin

**Affiliations:** 1 National Marine Fisheries Service, Southwest Fisheries Science Center, La Jolla, CA, USA

This article describes the sequencing and assembly of a reference genome for the Northern Bottlenose whale, and summarizes information about the data and assembly, all of which are publicly available. The introduction provides a description of what is known about the species and some information about conservation concerns, and potential applications of the genomic data.

 The only corrections I suggest are 1) description of what "BOLD barcode results" are, as they are not described in the paper or the linked web site, and 2) deletion of one copy of the sentence "Table 4 contains a list of relevant software tool versions and sources.", which is repeated before and after Table 4 in the "Evaluation of the final assembly" section.

 The legend for Figure 2 states that "The blue and pale-blue area around the outside of the plot shows the distribution of GC, AT and N percentages in the same bins as the inner plot." There appear to be three shades of blue, so what does the medium blue shaded region represent?

Are sufficient details of methods and materials provided to allow replication by others?

Yes

Is the rationale for creating the dataset(s) clearly described?

Yes

Are the datasets clearly presented in a useable and accessible format?

Partly

Are the protocols appropriate and is the work technically sound?

Yes

Reviewer Expertise:

Cetacean population genetics and genomics.

I confirm that I have read this submission and believe that I have an appropriate level of expertise to confirm that it is of an acceptable scientific standard.
